# Design of a Novel Equi-Biaxial Stretcher for Live Cellular and Subcellular Imaging

**DOI:** 10.1371/journal.pone.0140283

**Published:** 2015-10-14

**Authors:** Jasmin Imsirovic, Tyler J. Wellman, Jarred R. Mondoñedo, Elizabeth Bartolák-Suki, Béla Suki

**Affiliations:** Department of Biomedical Engineering, Boston University, Boston, Massachusetts, United States of America; Mayo Clinic College of Medicine, UNITED STATES

## Abstract

Cells in the body experience various mechanical stimuli that are often essential to proper cell function. In order to study the effects of mechanical stretch on cell function, several devices have been built to deliver cyclic stretch to cells; however, they are generally not practical for live cell imaging. We introduce a novel device that allows for live cell imaging, using either an upright or inverted microscope, during the delivery of cyclic stretch, which can vary in amplitude and frequency. The device delivers equi-biaxial strain to cells seeded on an elastic membrane via indentation of the membrane. Membrane area strain was calibrated to indenter depth and the device showed repeatable and accurate delivery of strain at the scale of individual cells. At the whole cell level, changes in intracellular calcium were measured at different membrane area strains, and showed an amplitude-dependent response. At the subcellular level, the mitochondrial network was imaged at increasing membrane area strains to demonstrate that stretch can lead to mitochondrial fission in lung fibroblasts. The device is a useful tool for studying transient as well as long-term mechanotransduction as it allows for simultaneous stretching and imaging of live cells in the presence of various chemical stimuli.

## Introduction

The *in vivo* mechanical environment of cells has been shown to influence normal cell function and has been implicated in a wide variety of diseases[1–3]. Cells are known to convert mechanical stimuli into biochemical responses, which are important for cell behavior and development. In particular, stretch plays an essential role *in vivo*, as seen in lung and cardiac development and surfactant secretion[2,4,5]. *In vitro* studies have shown that cell shape and orientation, dictated by the underlying cytoskeletal structure, is also influenced by stretch[6]. Likewise, calcium concentration within the cell responds to stretch, possibly through stretch-sensitive ion channels[7–9]. Thus, the effects of stretch are seen at time scales ranging from seconds to days, some of which are difficult to observe in real time.

Several types of mechanical stimuli are observed *in vivo* including tension, compression, shear and hydrostatic pressure, with some cells experiencing multiple stimuli simultaneously. In order to study the effects of such mechanical stimuli researchers have developed devices to deliver the appropriate type of mechanical stimuli in the appropriate dimensions[10]. For example, devices that deliver either uniaxial or biaxial stretch to cells by applying tension to an underlying membrane have been characterized in the literature[11–13]. The respiratory and cardiovascular systems are prime examples of cells experiencing cyclic stretch, which can be replicated by these devices. In particular, epithelial cells in lung alveoli and endothelial cells in large arteries experience stretch that can be well approximated with biaxial or equi-biaxial strains. Equi-biaxial cell stretching devices use either a vacuum driven system, such as the Flexercell FX-4000 Tension Plus System, or a motor driven indenter system to stretch silicone membranes[11,12]. These systems, however, either lack the ability to deliver cycle to cycle variability in stretch amplitude and frequency that is characteristic of blood pressure- and respiration-driven stretch *in vivo* or do not offer simultaneous imaging capability. Recently, a device using a moving magnet linear actuator was shown to deliver strains of arbitrary waveforms[14]. All three systems, however, were designed as large multi-well systems in order to stretch large numbers of cells at a time for biochemical analysis. While imaging can be performed on these multi-well systems, it requires either fixation of cells or for the samples to be taken out of the device.

A device which can simultaneously stretch and image cells would be beneficial in observing changes in intracellular structures due to stretch at shorter time scales concurrent with mechanical stimulation. Such a device would have to overcome several constraints such as size and specific design for use on a microscope stage. Likewise, high magnification objectives have small working distances, which constrain the dimensions of the device and site of cells within it. Upright and inverted microscopes image the cells from different sides, which may require a versatile design for an indenter system. In order to image cells the most important design consideration is to maintain cells in the field of view and in focus, which is complicated by the simultaneous stretching imposed by the device. Some cell stretching devices have been designed for use during microscopic observation but cannot deliver cyclic strain or are limited to uniaxial stretch[13]. A cyclic biaxial stretcher that allows microscopic observation does exist; however, it is limited to area strains up to ~30% and is configured for use with only inverted microscopes[15]. Furthermore, all of these devices use a lubricant to decrease friction between the membrane and an indenter, a technique that may decrease repeatability and accuracy.

Here we report the design and characterization of a device that can apply precision-controlled equi-biaxial stretch at the level of individual cells while simultaneously allowing live imaging of subcellular structures. To this end, we have designed, built and tested a single well device that deforms a membrane, on which cells are grown, via an indenter comprised of sixteen ball bearings that reduce friction. The device can be used simultaneously with both upright and inverted microscopes during stretching to image subcellular structures on time scales ranging from seconds to many hours. Furthermore the device can deliver precise changes in surface area with arbitrary waveforms and cycle to cycle variability due to the reproducible movement of the ball bearings. Using this device, we present examples of fluorescence microscopy to observe the effects of stretch on intracellular calcium levels and cellular structures such as the mitochondrial network.

## Materials and Methods

### Design of Cell Stretching Device

**Fig 1:**
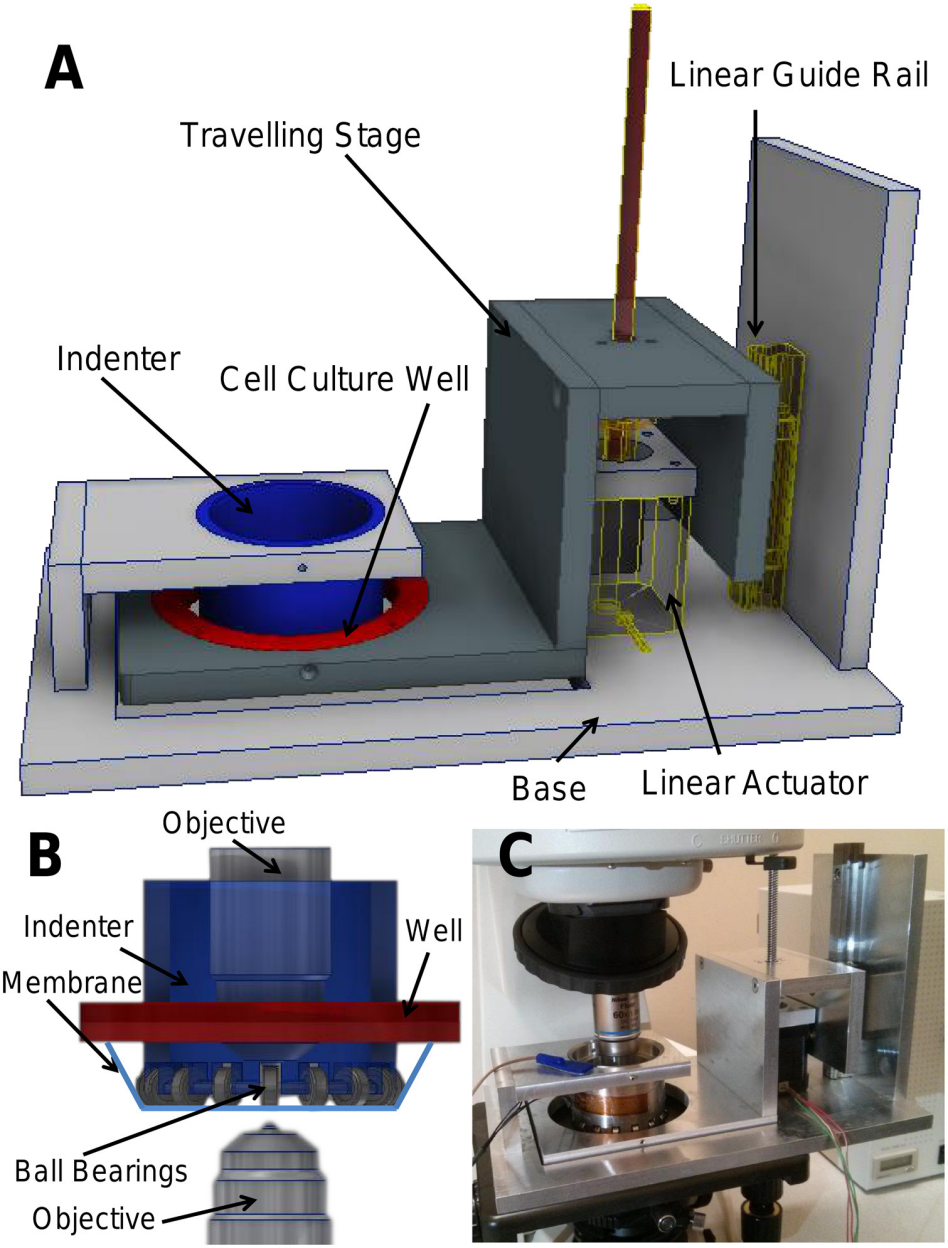
A side view of the biaxial stretching device (A). The device consists of a linear actuator that controls the vertical motion of a traveling stage, which has a custom well (red) with a silicone membrane (light blue) on which cells are seeded. Biaxial stretch is delivered to the cells by stretching the membrane over a hollow indenter (blue), which has ball bearings to reduce friction (B). Cells can be imaged either from above, by placing a water immersion objective inside the indenter, or from below using an inverted microscope. (C) An actual image of the system under an upright microscope.

A mechanical cell stretching device was developed to deliver biaxial strain while simultaneously imaging live cells (Fig 1A). The device delivers stretch to cells by deforming an optically clear elastic membrane, on which cells are seeded, over a post. The compact size and unique design of this device, however, allows it to be placed on a microscope stage to perform live cell imaging. Cells are cultured in a custom well comprised of stainless steel rings with a 0.127 mm thin silicone membrane bottom (Specialty Manufacturing Inc, Saginaw, MI). An insert limits the growing area for the cells to a circle of 10 cm^2^ at the center of the membrane. Three set screws secure the cell culture well to a traveling stage, which is actuated using a stepper motor linear actuator (Haydon Kerk, Waterbury, CT). The silicone membrane comes in contact with the cylindrical indenter post located directly above the cell culture well. The indenter post consists of sixteen stainless steel ball bearings (McMaster-Carr, Elhmhurst, IL) that minimize friction between the silicone membrane and the indenter, allowing for nearly frictionless sliding of the membrane around the indenter post and smooth deformation of the membrane. By controlling the vertical displacement of the traveling stage, biaxial deformation can be applied to the adherent cells. The temperature control system was adapted from an earlier open-dish incubator design, consisting of a temperature controller, thermocouple and the heating elements attached directly to the indenter[16].

Vertical displacement of the traveling stage is computer controlled via the movement of the stepper motor linear actuator. A custom Labview program was written to generate the appropriate step and direction pulses, which are output via a NI DAQ USB6221 (National Instruments, Austin, TX) to a stepper motor driver. After calibration of the device, arbitrary waveforms of varying amplitude (surface area strain) and frequency can be applied to the cells by controlling the traveling stage position. Imaging of the cells can be performed using both an inverted microscope, by imaging through the thin and transparent silicone membrane, or by an upright microscope, using a water-immersion lens (Fig 1B). The membrane remains in the same plane with minimal vertical displacement during stretching, fulfilling the primary design consideration. Thus live cells can be imaged during incremental changes in surface area.

### Calibration of the Device

To determine the relationship between the vertical displacement of the stage (indenter depth) and the change in surface area (ΔSA) of the membrane, we tracked the expansion and relaxation of a predetermined demarcated region on the membrane during quasi-static stretch. A circular region was defined in the center of the membrane using acrylic paint (Pēbēo, Cedex, France) to mark sixteen dots enclosing a circle with diameter 27 mm. To simulate the weight of media necessary for cell experiments, 10 mL of ddH_2_O was added to the membrane well. The acrylic dots were then imaged continuously during quasi-static stretch as the depth of the indenter was increased from 0 to 20 mm with short pauses at depth increments of 2 mm. Zero depth was defined as the point of first contact between the membrane and ball bearings of the indenter, where a spacer between the travelling stage and base was used to establish a datum point for all subsequent experiments. Custom image processing software (MATLAB, Mathworks, Natick, MA) was developed to segment the acrylic dots from the video recording and then track the change in area of the circular region they enclosed. The ΔSA was thus defined as the percent area change of the region at each incremental indenter depth. To determine whether the calibration changed over time, each membrane was stretched a total of 12 cycles with recordings acquired during cycle = 1, 2, 3, 6, 12. This was repeated for *n* = 3 different membranes such that the final calibration was determined by their average. Given this relationship between indenter depth and ΔSA, arbitrary strain waveforms could be generated with cycle-to-cycle variations.

To characterize the frequency response of the device, sinusoidal waveforms were generated according to the previously established calibration curve and used to stretch membranes at several frequencies and strain amplitudes. A circular region was again defined on the membrane using the acrylic dots that were tracked using the imaging software as before. Frequencies were logarithmically distributed over two decades between 0.01 and 1.00 Hz with area strain amplitudes of either 20% or 40% peak strain. Each membrane (*n* = 3) was stretched for 10 complete sinusoids for each frequency and amplitude. Separate quasi-static stretch calibrations, as described above, were performed before and after sinusoidal stretching to confirm that deformation of the membrane did not occur subsequent to repeated stretching.

To confirm that the strain applied at the length scale of the entire membrane well (ie, macrostrain) was accurately translated to the strain experienced at the length scale of individual cells (i.e, microstrain), the membrane was first coated using Nile Red fluorescent high intensity beads (Spherotech, Lake Forest, IL) with diameters of 2.27 and 5.1 μm. Using the previously established ΔSA-indenter depth relationship, a prescribed macrostrain increasing from 0 to 40% strain was applied to the entire membrane while images of the beads were acquired during static stretch at increasing intervals of either 2.5% or 5% strain using a Nikon Eclipse 50i fluorescence microscope with a Nikon Fluor 60X water immersion objective. From these images, some of the 2.27 μm beads were selected to be the vertices of a polygon and tracked at each strain level such that the ΔSA of the defined polygon was used to compute the local micro strain. This procedure was then repeated at 9 different locations from 2 separate membranes to compare the measured micro strain to the prescribed macrostrain.

Finally, to verify that the biaxial stretch was uniform across the entire seeding area, a square consisting of 13 dots was marked on the silicone membrane. The membrane area strain was recorded at increasing levels of indenter depth. The centroid of each dot was determined and used to construct a triangular mesh between the dots. The change in area of each triangle was measured and compared to the prescribed change in area.

### Cell Culture and Live Cell Imaging

To demonstrate cell adherence and viability in the custom wells, primary bovine lung fibroblasts were cultured on the silicone membranes at the first passage. Primary lung fibroblasts were isolated according to the explant isolation technique [17] from bovine lungs supplied by Research 87 (Boylston, MA). Briefly, a sample of the lung was dissected and the pleura was removed along with any large airways. The parenchyma was cut into small pieces and placed into a flask with Dulbecco’s Modified Eagle Medium (DMEM) (Life Technologies) supplemented with 10% fetal bovine serum (Life Technologies), 0.1 μg/ml primocin (Invivogen), 10 ml/L penicillin-streptomycin (Sigma-Aldrich), 2.5 μg/ml fungizone (Life Technologies). Explants were removed following cell outgrowth a week after isolation. The silicone membrane wells were assembled and sterilized using alcohol and UV light exposure for 24 hours. Prior to cell seeding, the membrane was coated to an area of 10 cm^2^ in the middle of the well using 25 μg/ml bovine fibronectin (Sigma-Aldrich). Cells were seeded on the membrane at a density of 2x10^5^ cells/cm^2^ inside an insert of area 10 cm^2^ and cultured in supplemented DMEM described above. After adhering, cells were incubated with fluorescent dyes that stained the cytosol, the mitochondria, or intracellular calcium as examples of live cell imaging. The cell cytosol was stained using 5 μM CellTracker Green (Life Technologies), incubating for 45 minutes and washing before imaging. Similarly, tetramethylrhodamine methyl ester (TMRM) (Life Technologies) was used to stain active mitochondria with 300 nM concentration and incubation for 45 minutes. Individual cells were tracked and images were taken using a Nikon Eclipse 50i widefield microscope with a Nikon Fluor 60X water immersion objective (NA = 1.0,) as area strain was increased from zero to 40%. This imaging setup had a lateral resolution of 0.285 μm and a field of depth of 750 nm approximately. Calcium imaging was performed using the Fluo-4 Direct Assay Kit (Life Technologies) according to the manufacturer’s specifications. The appropriate working solution was prepared and cells were incubated for one hour at 37°C before imaging. Cells were imaged using an Olympus IX81 widefield microscope with an Olympus LUCPlanFL N 20X objective (NA = 0.45) and 494 nm and 515 nm excitation and emission filters for Fluo-4, respectively. This imaging setup had a lateral resolution of 570 nm and a field of depth of 2.5 μm approximately. Cells were first imaged for 10 minutes with no stimulus to determine a baseline of the calcium signal, followed by a single sinusoidal stretch of either 3% or 10% area strain lasting 6 seconds. Cells were then imaged for 10 minutes to observe transient calcium changes. A subset of experiments was extended to include a 5 minute cyclic sinusoidal stretch at 10% area strain. To test the cell viability and the response of the calcium signal to an inhibitor, the L-type calcium channel blocker verapamil, which may also affect the mechanosensitive calcium channels, was used after the baseline imaging and was followed by a single stretch and a 5 minute cyclic stretch period with 10 minutes of imaging between each stimulus. To test the ability of our system to study long-term effects of stretch, calcium imaging was also performed using Fura2AM (Life Technologies) ratiometric dye using excitation filters at 340 and 380 nm and an emission filter 515 nm. Cells were incubated with 5 μM of Fura2AM for 45 minutes at 37°C followed by 30 minutes at room temperature. Cells were then washed with fresh media and 2.5 mM of probenecid was added to prevent sequestration of Fura2AM. Cells were then stretched for 60 minutes at 10 cycles per minute, mimicking bovine breathing frequency, up to 10% area strain while images of the same field of view were acquired at the 0, 5, 20 and 60 minute time points. Ratiometric images were obtained by dividing the 340 nm excitation images by the corresponding 380 nm images for each cell.

### Statistical Analysis

Data are presented as mean ± standard deviation for each group. Data were analyzed using one-way ANOVA and differences between groups were considered statistically different for p < 0.05.

## Results

### Device Characterization

**Fig 2:**
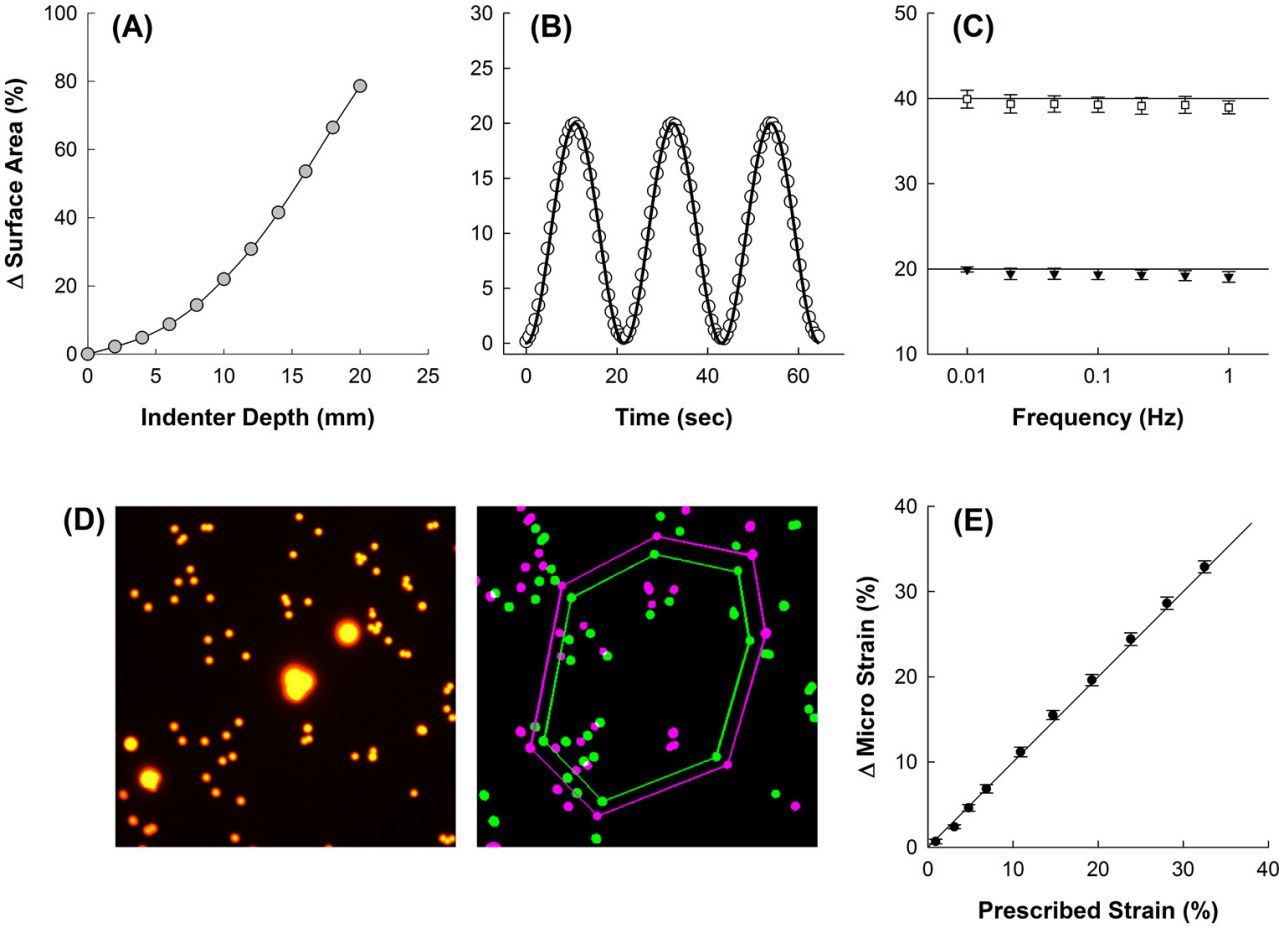
(*A*) Nonlinear relationship between the indenter depth and the corresponding change in surface area of a demarcated region on the membrane; this calibration curve was subsequently used to prescribe strain waveforms as a function of indenter depth. Data points represent the average of n = 3 calibrations with different membranes; standard deviation bars (not shown) are smaller than symbols. (*B*) Representative sinusoidal waveform applied to membrane (amplitude, 20% strain; frequency, 0.0464 Hz), the input waveform (solid line) and the observed change in surface area (open circles) are shown demonstrating close agreement between input and measurement. (*C*) Frequency response of the device is flat for stretch frequencies between 0.01–1.0 Hz; sinusoidal waveforms with prescribed strain amplitudes of 20% (filled triangles) and 40% (open squares) are shown with reference lines, error bars represent the standard deviations (N = 3). (*D*) *Left*: characteristic image of marker beads used to track micro strain; *Right*: detection algorithm showing beads at baseline (green) and after applied strain (magenta), specific beads are tracked and the areas enclosed by their polygons are used to determine the corresponding micro strain. (*E*) Change in micro strain closely follows the prescribed macrostrain, line of identity is shown for reference; error bars represent standard deviations (n = 9).

Fig 2A shows the nonlinear relationship between indenter depth and ΔSA measured at 2 mm increments from 0 to 20 mm of indenter depth. Data points represent the average of three separate calibrations with different membranes. Standard deviations were too small to be plotted, with the largest standard deviation being 1.3%. The unloading curve was also measured with a hysteresis between loading and unloading of only 2.9% (not shown). These data demonstrate that the system is capable of reproducibly delivering strains of up to 80% across several cycles for a single membrane as well as between different calibrations. Given this relationship between indenter depth and ΔSA, it is possible to generate stretch waveforms with prescribed strain amplitudes. For example, the prescribed and observed waveforms for a sinusoid with frequency 0.0464 Hz and strain amplitude 20% strain are shown in Fig 2B. The prescribed and delivered waveforms were nearly identical in shape, amplitude and frequency demonstrating the ability of the device to reproduce an arbitrary area strain waveform. A similar behavior was observed across several of frequencies and strain amplitudes. Fig 2C shows that the device has a flat frequency response over the range of 0.01 to 1.00 Hz at both 20% and 40% area strain.

We also demonstrated that the micro strain measured at the length scale of individual cells matched the prescribed macrostrain applied at the length scale of the entire membrane. The local micro strain was measured by imaging beads (Fig 2D, left) that were used to define the vertices of a polygon (Fig 2D, right) at baseline (green) and after a prescribed macrostrain was applied (magenta). The mean and standard deviation of the micro strain sampled at 9 different locations from two separate membranes are compared to the prescribed macrostrain in Fig 2E, and observed to closely follow the line of identity. The maximum standard deviation in microstrain for any prescribed macrostrain was found to be 0.75%.

**Fig 3:**
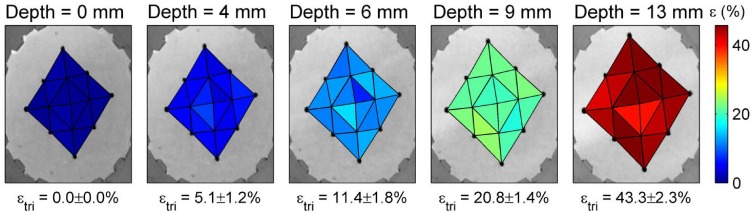
Change in surface area of sixteen triangles on the membrane shows the regional area strains at indenter depths of 0, 4, 6, 9 and 13 mm, with ΔSA ranging from 0% (blue) to 45% (red). The average and standard deviation of ΔSA for all triangles is shown at each depth. The regional deformation is consistent for all triangles with a maximum standard deviation of 2.3% at the highest observed indenter depth.

Fig 3 shows the regional ΔSA of individual triangles, ranging from 0% strain (blue) to 45% (red). As shown, the ΔSA of individual triangles are all within 2.3% area strain, which may be within the error of measurement. Thus the device delivers uniform and repeatable equi-biaxial strain to the membrane.

### Cell Deformation

Bovine fibroblasts grown on fibronectin coated silicone membranes were observed under the microscope for two hours within the device and showed normal morphology and no change in cell viability. Thus, the temperature control system and HEPES buffered media were sufficient for observing cells during longer-term experiments. Imaging sometimes required minor refocusing because the silicone membrane would shift up and down relative to the objective as the membrane was stretched to higher area strains. This is due to the added tension in the silicone membrane and the weight of the media being shifted around at different strains.

**Fig 4:**
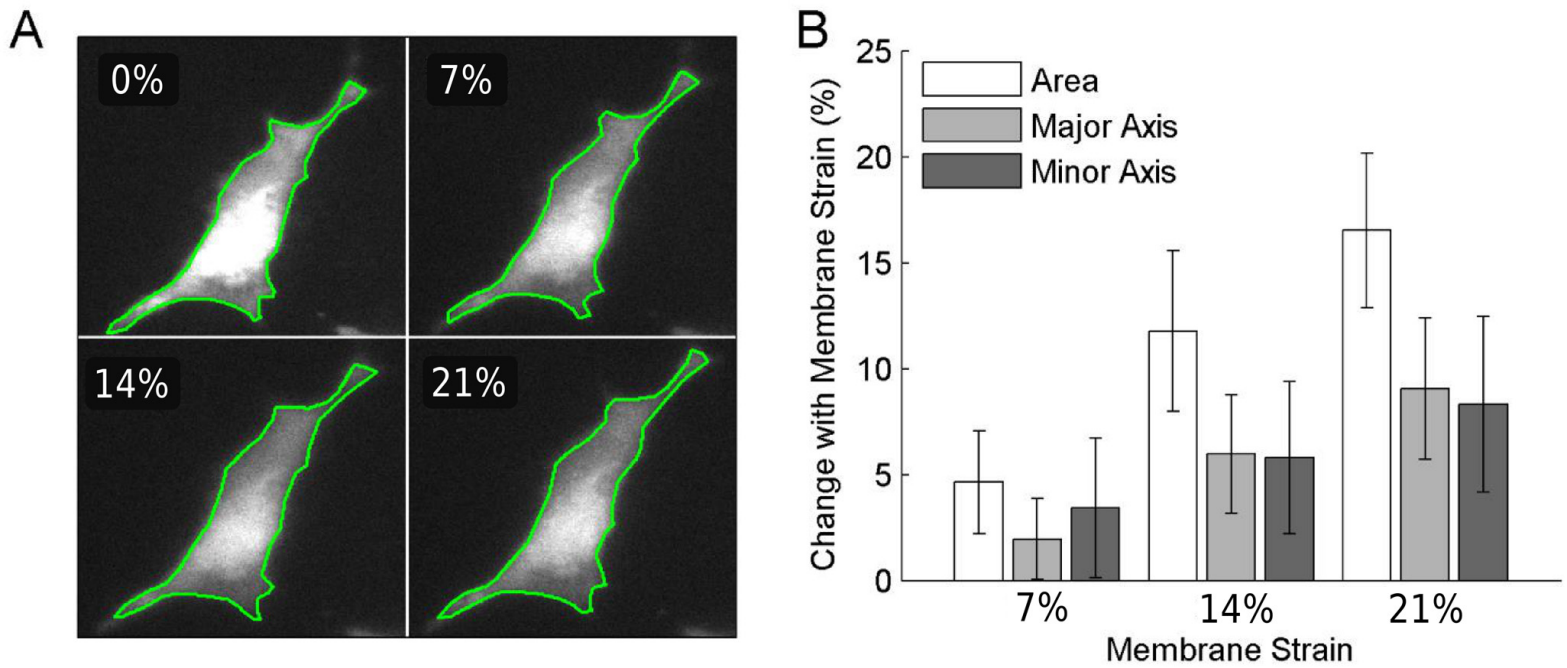
An example of a cell tracked and imaged in increments of 7% area strain (A). The outline of the cell was manually selected and the cell shape was fitted with an ellipse whose major and minor axes were measured (B). The area strain of cells was measured (n = 8) and compared to the prescribed membrane area strain. The measured cell area strain is lower than the prescribed strain, but is within one standard deviation at each measured increment. The major and minor axes of the cell increase equally, demonstrating biaxial strain.

**Fig 5:**
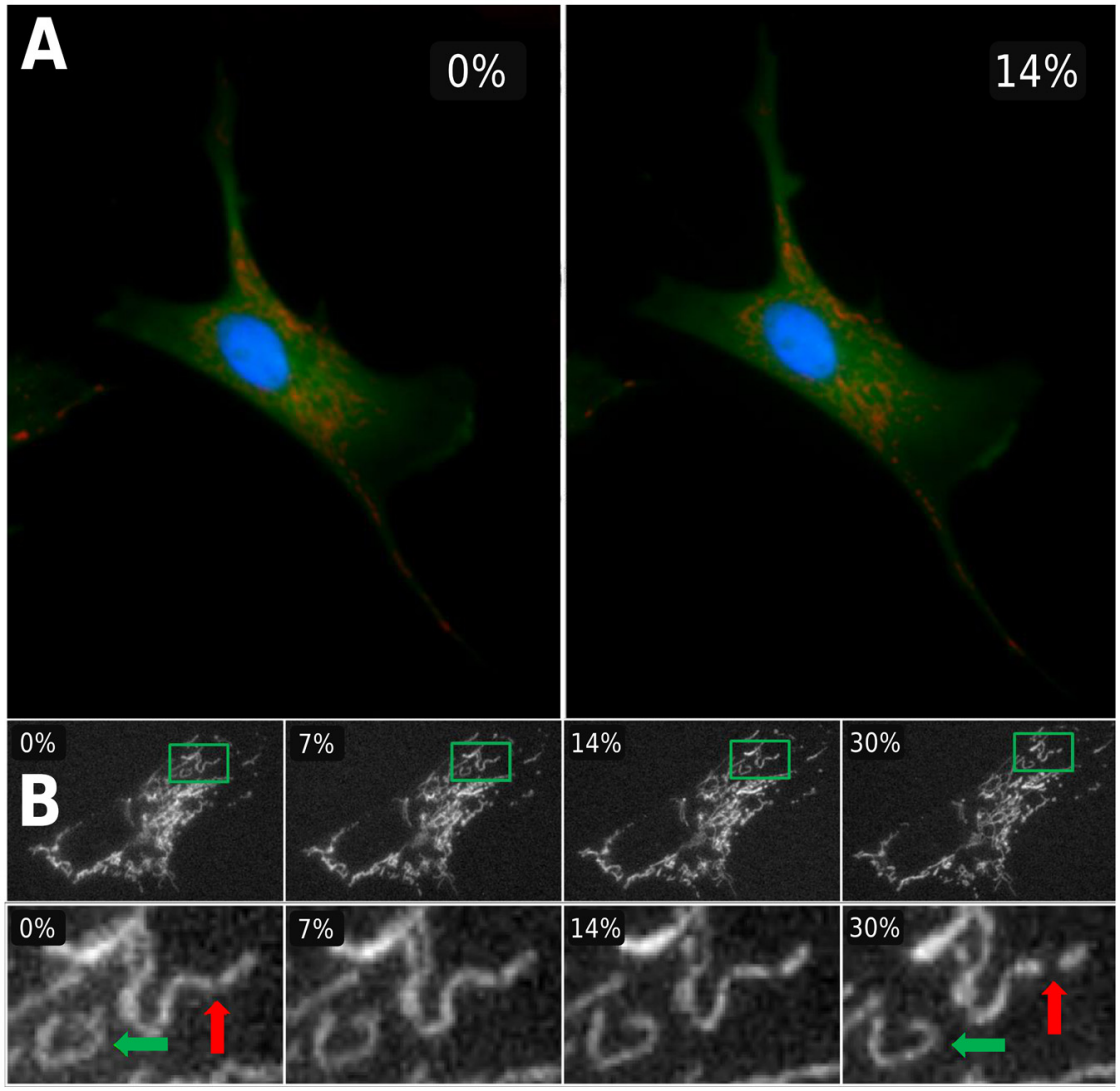
(A) A cell labeled for cytosol (green), mitochondria (red) and nucleus (blue) at 0% (left) and 14% (right) membrane area strains. (B)The top row shows the mitochondrial network of an entire cell imaged during constant strain application at increments of 0, 10, 20 and 40% change in membrane surface area. The bottom row shows a detail of an individual cluster (the green rectangle in top row) changing shape as higher strains are applied (green arrow), as well as a cluster undergoing fission and splitting into two smaller clusters (red arrow).

Fig 4A shows an example of a manually segmented cell at increasing levels of area strain. Cells were labeled with CellTracker Green to delineate the area of the cells. The mean and standard deviation of change in cell area at various levels of membrane strain was quantified in 8 cells. In addition, cell areas were approximated as an ellipse, and strains of the major and minor axes were also measured (Fig 4B). The measured change in cell area increases linearly and is within one standard deviation of the prescribed area strain; however, the measured cell strain is consistently lower than the prescribed change in membrane area. Fig 5A shows a cell with labeled nucleus, cytosol and mitochondria imaged at 0% strain and 14% strain. Fig 5B exemplifies how the mitochondrial network stretches in a bovine fibroblast captured with a 60X objective at 0, 7, 14, and 30% strain. The top row shows the network of the entire cell deforming during stretch, while the bottom row shows a detail of individual clusters within the cell. A cluster can be seen changing shape as higher strains are applied (green arrow), while another cluster may undergo stretch-induced fission by splitting into two smaller clusters (red arrow).

### Calcium Imaging

**Fig 6:**
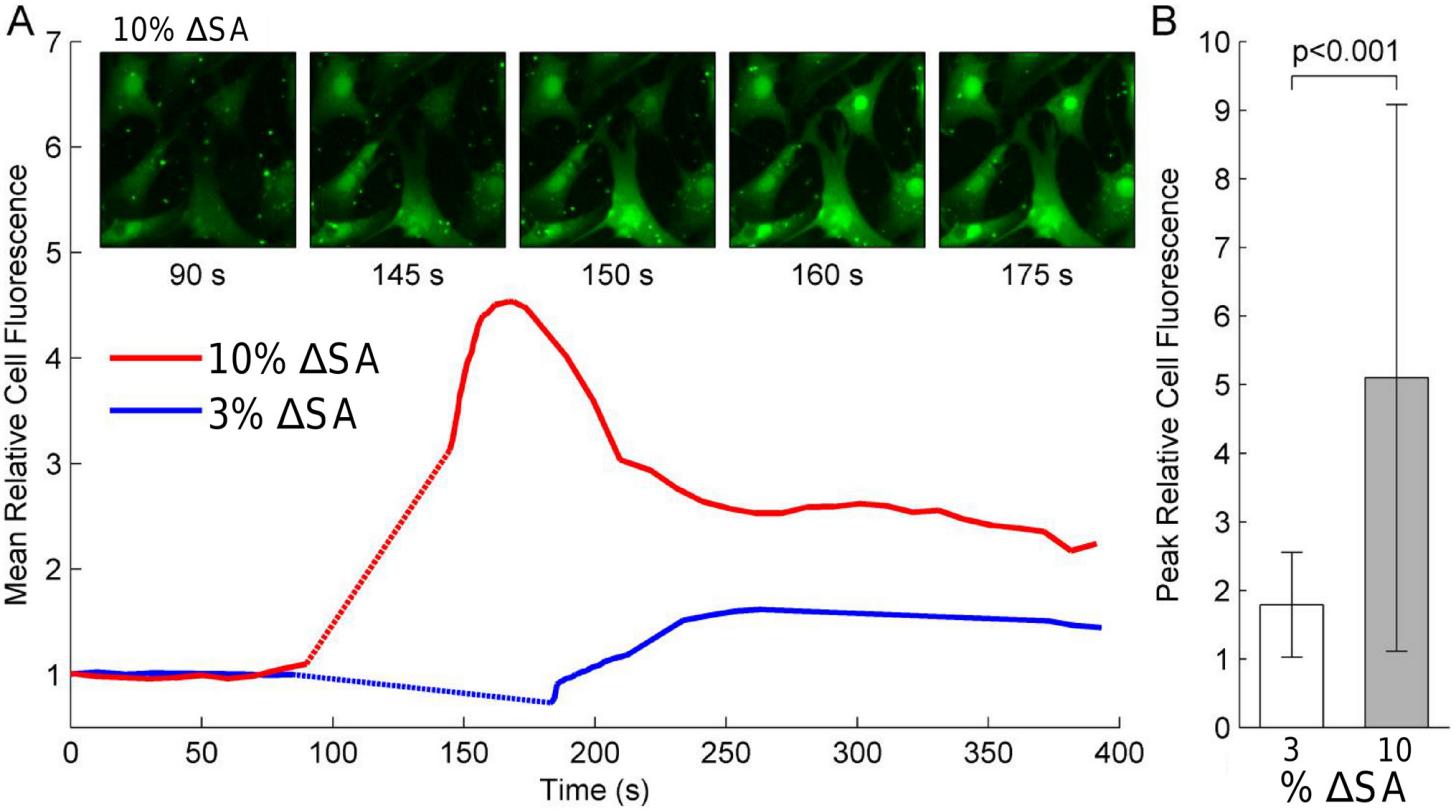
Intracellular calcium level responses of primary bovine fibroblasts to single sinusoidal equi-biaxial strains. Cells were loaded with Fluo4 calcium indicator dye and subjected to strains of 3 or 10% change in membrane surface area (ΔSA). (A) Following single 6 s long stretch (dotted line), cells stretched to 10% ΔSA showed large transient increases in fluorescence relative to baseline (mean of 77 cells), while cells stretched to 3% ΔSA showed little response (mean of 44 cells). Images of cells stretched to 10% ΔSA revealed differences between neighboring cells in the timing of their response to stretch, with some cells responding immediately and other requiring up to 10 s to respond. (B) The changes in fluorescence were significantly greater with 10% ΔSA than with 3% ΔSA (p<0.001).

**Fig 7:**
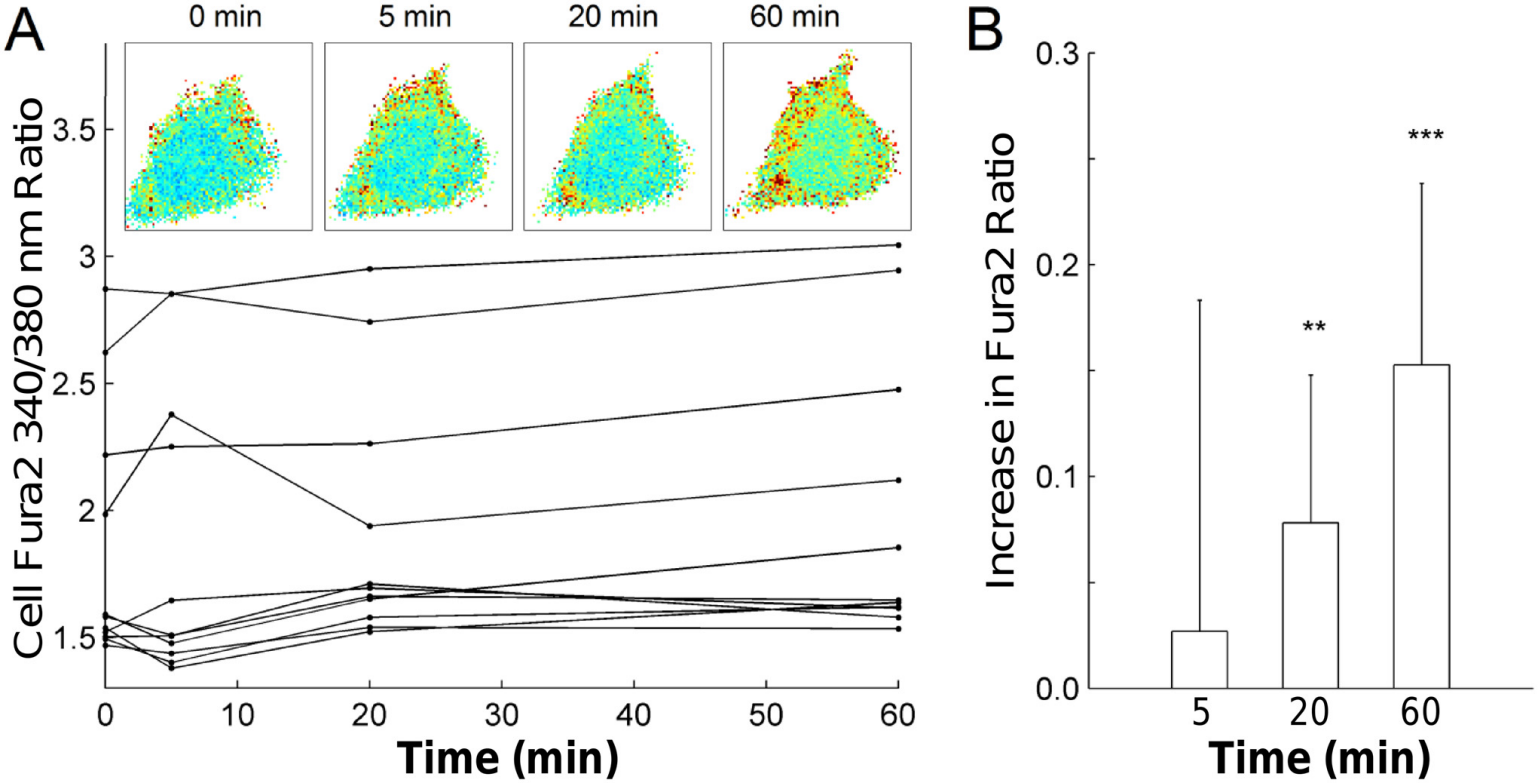
Response of intracellular calcium levels in bovine fibroblasts up to one hour of continuous sinusoidal stretching with maximum amplitude of 10% change in membrane surface area. Average ratios of Fura2 fluorescence at 340 and 380 nm excitation wavelengths were computed in each of 11 cells. Individual cell responses varied (A), but there was an overall trend of increasing Fura2 ratios over time, with significant increases present at 20 and 60 min relative to baseline (B). **, p<0.01; ***, p<0.001

Fig 6A shows the mean intensity of Fluo4 calcium indicator dye for cells stretched to 3% (n = 44) and 10% (n = 77) membrane area strain. Following a single stretch to 10% ΔSA, large transient increases in signal intensity with respect to baseline were observed within individual cells. Cells stretched to 3% membrane area strain showed smaller increases, with peak relative cell fluorescence significantly greater for 10% membrane area strain than for 3% membrane area strain (p < 0.001) (Fig 6B). Of note, some cells demonstrated immediate responses to stretch, while others exhibited a delay in response of up to 10 seconds. Cells stretched cyclically at 10% area strain for 5 minutes (n = 45) showed a higher increase in fluorescence relative to baseline after the stretch (35% ± 21%) versus those treated with verapamil (17% ± 7%) (n = 49) at the outset of the experiment (p<0.001) (data not shown). Interestingly, there was no difference in response immediately after the cessation of cyclic stretch; instead, there was a delayed response of the cells after cyclic stretch. Likewise Fig 7A shows the ratio of Fura2AM fluorescence at 340 and 380 nm excitation wavelengths for 11 cells stretched continuously with a cyclic sinusoidal waveform of maximum 10% area strain. Relative to baseline levels, the ratio levels increased at 20 minutes (p < 0.01) and at 60 minutes (p<0.001) (Fig 7B).

## Discussion

### Device Characteristics

The device presented was designed to deliver equi-biaxial stretch while simultaneously imaging live cells. Most current devices are not designed for live imaging, thus researchers often fix cells in order to visualize them, which can introduce artifacts and prohibit studying dynamics. Our novel design includes several important features that have not yet been combined in previous biaxial stretchers including: a) the ability to cyclically stretch cell substratum with programmable, cyclic or arbitrary waveforms that can vary from cycle to cycle; b) a highly accurate and repeatable ΔSA at the scale of single cells and at all levels of applied strain as detailed by the calibration; c) the ability to deliver physiological and supra-physiological levels of uniform equi-biaxial strain; d) size and versatility that allow for imaging using either an upright or inverted microscope with high magnification objectives; and e) a temperature control system and the use of buffered media to allow long-term observation of cells during stretch.

We have shown that the device applies uniform equi-biaxial stretch to custom wells with silicone membranes. By characterizing the relationship between membrane area changes and travel of the linear actuator we are able to deliver customized waveforms of area strain to adherent cells. Furthermore, the calibration validates the 16 ball bearing indenter as a novel, precise and accurate method for imposing equi-biaxial strain. The maximum standard deviation reported for the macrostrain and microstrain calibrations are 1.3% and 0.75% respectively, which are amongst the lowest values for published biaxial stretching systems. Likewise, the macrostrain calibration shows only 2.9% hysteresis, which suggests that the silicone membrane is elastic and that the friction in the bearings is low. The hysteresis could not be compared to other systems as it is usually not reported. Nevertheless, the precise and accurate strains delivered by this system compare favorably to existing devices and are likely due to the reproducible movement of the ball bearings. Indeed, earlier designs without the ball bearings often resulted in dry friction between the membrane and the indenter with less reproducible micro strains.

It is important to emphasize the importance of a fixed point on the device that can be used as a datum for all calibrations and experiments. Devices that rely on indentation of a membrane by a circular post will inherently have nonlinear ΔSA-depth relationships due to their geometry. Considering this nonlinearity, small errors in the starting position of the membrane will produce significant errors at higher strains. Initially, the depth at which the membrane contacted the ball bearings was taken as zero depth and a spacer was machined to go between the base and the travelling stage, allowing that depth to be reproducibly visited. After performing both the macrostrain and microstrain calibrations a discrepancy was noticed between the two; while the two calibration curves had the same curvature, there was an offset of only ~1.4 mm in depth between them accounting for an error up to 6% ΔSA at a strain of 20%, a margin of error of 30%. Thus the nonlinearity of the ΔSA in such devices must be well characterized and it is not accurate for the user to set the initial position by feel as reported elsewhere[15]. The calibration must also be done in the presence of fluid since the weight of the fluid also shifts the calibration curve.

Unlike in pressure driven devices[10], the silicone membrane stays in the same plane during stretch, addressing one of the main design considerations. However, the membrane is not completely immobile and travels up and down in small increments as strain is applied. Thus, refocusing is sometimes required when tracking a cell through increasing levels of strain. Importantly, the cells return to the same imaging plane if they are brought back to zero or any initial level of strain, allowing for rapid and automated imaging of cells at a fixed strain or following cessation of cyclic stretch. A possible solution to cells traveling out of the focal plane is the use of three dimensional imaging with a confocal microscope. Since refocusing requires human input and a variable amount of exposure time, 3D imaging could capture the cells as they travel outside of the focal plane with repeatable exposures. For small strains and small regions this technique should be rapid and feasible, while larger strains can also be accommodated by measuring the vertical displacement caused by stretch initially and entering the necessary vertical position for all subsequent stretches. A calibration could also be performed to account for vertical displacement for larger strains to determine the initial position of the z-stack. This approach would not only solve the issue of refocusing but also provide more information about the cell, such as cell height and the vertical positioning and travel of organelles.

Compared to other biaxial stretchers, we introduce the ability to simultaneously image from above and below, using a dual microscope. A water immersion objective allows high magnification imaging from above to better study the dynamics of subcellular structures, while the membrane is thin enough to allow imaging from below with magnification as high as 40x. Thus, two imaging modalities can be used in concert with biaxial stretching using this versatile design. Furthermore, physiological and supra-physiological strains, such as those observed in diseased and pathological conditions as in acute lung injury[18], can be applied to the cells with membrane deformations as high as 80% area strain. Finally, we demonstrate the ability to track a single cell through increasing levels of area strain while observing several intracellular structures.

### Cell Deformation and Mitochondria

The cell area changes do not perfectly reflect the prescribed membrane area changes (Fig 4), as reported for other biaxial stretching devices. Specifically, Tschumperlin et al. showed that the cell area changes are nearly identical to substrate area changes[11]. While the measured cell area strain is consistently below the prescribed substrate area strain, it is still within one standard deviation of the true substrate area strain. Several factors could be responsible for this minor discrepancy. Firstly, the type of cells used by Tschumperlin et al. were type II epithelial cells, which have a circular morphology and are cultured at a high density such that neighboring cells are in close contact with each other[11]. Conversely, our study observed cells that are contractile with a dipole morphology that were seeded more sparsely, such that cells responded independently to strain rather than as an interconnected sheet attached to the membrane. Thus, the microscopic deformations at the cell level were likely more dependent on the Poisson ratio of each cell and how it was attached to the membrane. It is possible that fibroblasts at higher strain partially released their attachment to the membrane or the elasticity of the attachment itself takes up some strain. Additionally, the manual segmentation of the cell border was an approximation of cell area, particularly with very thin protrusions found in fibroblast cells. The changes in the major and minor axes fit to the segmented cells were nearly identical and therefore the cell experienced biaxial strain, despite not reaching the prescribed membrane area strain. Considering that each cell is imaged individually, the true cell area strain can be measured from images rather than assuming a prescribed membrane area strain.

For mechanobiological studies, the ability to measure the true cell area strain for each cell can be crucial, since cell morphology and attachment inevitably influence the strain that an adherent cell ultimately experiences. Relating individual cell strains to biological responses may help better understand cellular processes dependent on strain amplitude such as signaling or cytoskeletal rearrangement. For example, the response of the mitochondrial network at different strains demonstrates the utility of the stretching device as a tool for studying the effects of stretch on subcellular structures. Mitochondrial network structure is known to affect cell function with respect to energy production as well as being a predictor of apoptosis[19,20]. Recently, actin has been found to play a role in mitochondrial fission, which regulates the network itself[21]. Furthermore, both monotonous cyclic and cycle by cycle variable stretches can cause reorganization of the mitochondrial network and changes in mitochondrial membrane potential[1,22,23]. Likewise, our results suggest that cell deformation can induce mitochondrial deformation as well as fission (Fig 5B).

### Calcium Imaging

Calcium signaling is a ubiquitous signaling pathway in cells that is regulated by many factors[24]. The influx of extracellular calcium ions (Ca^2+^) often leads to a release from internal stores. Ca^2+^ influx can be regulated by voltage-dependent Ca^2+^ channels, receptor-operated Ca^2+^ entry, store-operated Ca^2+^ entry, and by stretch-activated cation channels[8]. Considering that Ca^2+^ is involved in numerous functions of the cell, such as muscle contractions, enzyme activity and cytoskeletal reorganization, stretch-induced regulation of intracellular Ca^2+^ influences many fundamental cell functions[24]. Using our new device, we demonstrated the stretch-dependence of intracellular Ca^2+^ in bovine lung fibroblasts. Importantly, a single stretch of 10% membrane strain caused a large transient increase in intracellular Ca^2+^, while a stretch of 3% showed a significantly smaller response using the Fluo4 Ca^2+^ indicator (Fig 6). Cyclic stretch also proved to be a stimulator of a cellular calcium response, which was decreased by the introduction of verapamil. Verapamil is known to affect the L-type voltage gated calcium channels, and may also affect mechanosensitive calcium channels [25]. More persistent changes in intracellular Ca^2+^ were measured using the Fura2AM ratiometric dye, which showed that intracellular Ca^2+^ levels increased significantly after just 20 minutes of continuous cyclic stretch and continued to increase after 60 minutes of stretch (Fig 7). Using a smaller magnification objective (20X) allows for a larger field of view and thus the calcium levels can be tracked in multiple cells at once. Using multiple live cell dyes simultaneously, such as the Fluo4 with TMRM, can allow for concurrent measurement of calcium levels and mitochondrial membrane potential.

## Conclusion

In summary, we present a novel equi-biaxial cell stretching device that provides accurate changes in cell substrate area during high magnification microscopic imaging. This device is a versatile research tool that can deliver strains ranging from small precise physiological strains to supra-physiological strains found in pathological conditions. We demonstrated the versatility of the device by observing the mitochondrial network and calcium imaging, on time scales ranging from seconds to hours. This device provides unique capabilities to study mechanotransduction mechanisms via imaging modalities.

## References

[1] Bartolák-Suki E, Imsirovic J, Parameswaran H, Wellman TJ, Martinez N, Allen PG, et al Fluctuation-driven mechanotransduction regulates mitochondrial-network structure and function. Nat Mater. 2015; 10.1038/nmat4358 26213900

[2] Ingber DE. Cellular mechanotransduction: putting all the pieces together again. FASEB J. 2006;20: 811–27. 10.1096/fj.05-5424rev 16675838

[3] Janmey P a, Miller RT. Mechanisms of mechanical signaling in development and disease. J Cell Sci. 2011;124: 9–18. 10.1242/jcs.071001 21172819 PMC3001405

[4] Wirtz HR, Dobbs LG. Calcium mobilization and exocytosis after one mechanical stretch of lung epithelial cells. Science. 1990;250: 1266–9. 2173861 10.1126/science.2173861

[5] Arold SP, Bartolák-Suki E, Suki B. Variable stretch pattern enhances surfactant secretion in alveolar type II cells in culture. Am J Physiol Lung Cell Mol Physiol. 2009;296: L574–81. 10.1152/ajplung.90454.2008 19136581 PMC2670764

[6] Greiner AM, Chen H, Spatz JP, Kemkemer R. Cyclic tensile strain controls cell shape and directs actin stress fiber formation and focal adhesion alignment in spreading cells. PLoS One. 2013;8: e77328 10.1371/journal.pone.0077328 24204809 PMC3810461

[7] Ito S, Suki B, Kume H, Numaguchi Y, Ishii M, Iwaki M, et al Actin cytoskeleton regulates stretch-activated Ca2+ influx in human pulmonary microvascular endothelial cells. Am J Respir Cell Mol Biol. 2010;43: 26–34. 10.1165/rcmb.2009-0073OC 19648475 PMC2911568

[8] Ito S, Kume H, Naruse K, Kondo M, Takeda N, Iwata S, et al A novel Ca2+ influx pathway activated by mechanical stretch in human airway smooth muscle cells. Am J Respir Cell Mol Biol. 2008;38: 407–13. 10.1165/rcmb.2007-0259OC 17975175

[9] Mohanty MJ, Li X. Stretch-induced Ca(2+) release via an IP(3)-insensitive Ca(2+) channel. Am J Physiol Cell Physiol. 2002;283: C456–62. 10.1152/ajpcell.00057.2002 12107055

[10] Brown TD. Techniques for mechanical stimulation of cells in vitro: a review. J Biomech. 2000;33: 3–14. 10609513 10.1016/s0021-9290(99)00177-3

[11] Tschumperlin DJ, Margulies SS. Equibiaxial deformation-induced injury of alveolar epithelial cells in vitro. Am J Physiol. 1998;275: L1173–83. 9843855 10.1152/ajplung.1998.275.6.L1173

[12] Arold SP, Wong JY, Suki B. Design of a new stretching apparatus and the effects of cyclic strain and substratum on mouse lung epithelial-12 cells. Ann Biomed Eng. 2007;35: 1156–64. 10.1007/s10439-007-9262-5 17578668

[13] Gerstmair A, Fois G, Innerbichler S, Dietl P, Felder E. A device for simultaneous live cell imaging during uni-axial mechanical strain or compression. J Appl Physiol. 2009;107: 613–620. 10.1152/japplphysiol.00012.2009 19498100

[14] Lau JJ, Wang RM, Black LD. Development of an arbitrary waveform membrane stretcher for dynamic cell culture. Ann Biomed Eng. 2014;42: 1062–73. 10.1007/s10439-014-0976-x 24473700 PMC3976041

[15] Huang L, Mathieu PS, Helmke BP. A stretching device for high-resolution live-cell imaging. Ann Biomed Eng. 2010;38: 1728–40. 10.1007/s10439-010-9968-7 20195762 PMC3468334

[16] Heidemann SR, Lamoureux P, Ngo K, Reynolds M, Buxbaum RE. Open-dish incubator for live cell imaging with an inverted microscope. Biotechniques. 2003;35: 708–14, 716 14579735 10.2144/03354bi01

[17] Baglole CJ, Reddy SY, Pollock SJ, Feldon SE, Sime PJ, Smith TJ, et al Isolation and Phenotypic Characterization of Lung Fibroblasts Fibrosis Research. Totowa, NJ: Humana Press; 2005 pp. 115–127. 10.1385/1-59259-940-0:115 16118449

[18] Uhlig U, Uhlig S. Ventilation-Induced Lung Injury Comprehensive Physiology. Hoboken, NJ, USA: John Wiley & Sons, Inc.; 2011 10.1002/cphy.c100004 23737198

[19] McBride HM, Neuspiel M, Wasiak S. Mitochondria: more than just a powerhouse. Curr Biol. 2006;16: R551–60. 10.1016/j.cub.2006.06.054 16860735

[20] Youle RJ, Karbowski M. Mitochondrial fission in apoptosis. Nat Rev Mol Cell Biol. 2005;6: 657–63. 10.1038/nrm1697 16025099

[21] Korobova F, Ramabhadran V, Higgs HN. An actin-dependent step in mitochondrial fission mediated by the ER-associated formin INF2. Science. 2013;339: 464–7. 10.1126/science.1228360 23349293 PMC3843506

[22] Chapman KE, Sinclair SE, Zhuang D, Hassid A, Desai LP, Waters CM. Cyclic mechanical strain increases reactive oxygen species production in pulmonary epithelial cells. Am J Physiol Lung Cell Mol Physiol. 2005;289: L834–41. 10.1152/ajplung.00069.2005 15964900

[23] Shinmura A, Tsukamoto A, Hamada T, Takemura K, Ushida T, Tada S. Morphological Dynamics of Mitochondria in Bovine Aortic Endothelial Cell under Cyclic Stretch. Adv Biomed Eng. 2015;4: 60–66. 10.14326/abe.4.60

[24] Clapham DE. Calcium signaling. Cell. 2007;131: 1047–58. 10.1016/j.cell.2007.11.028 18083096

[25] Quill B, Irnaten M, Docherty NG, McElnea EM, Wallace DM, Clark a. F, et al Calcium channel blockade reduces mechanical strain-induced extracellular matrix gene response in lamina cribrosa cells. Br J Ophthalmol. 2015;99: 1009–1014. 10.1136/bjophthalmol-2014-306093 25795916

